# REDECA: A Novel Framework to Review Artificial Intelligence and Its Applications in Occupational Safety and Health

**DOI:** 10.3390/ijerph18136705

**Published:** 2021-06-22

**Authors:** Maryam Pishgar, Salah Fuad Issa, Margaret Sietsema, Preethi Pratap, Houshang Darabi

**Affiliations:** 1 Mechanical and Industrial Engineering, University of Illinois at Chicago, Chicago, IL 60609, USA; mpishg2@uic.edu; 2 Agricultural and Biological Engineering, University of Illinois at Urbana-Champaign, Urbana, IL 61801, USA; salah01@illinois.edu; 3 Environmental and Occupational Health Sciences, University of Illinois at Chicago, Chicago, IL 60612, USA; msiets2@uic.edu (M.S.); plakshmi@uic.edu (P.P.)

**Keywords:** artificial intelligence, worker health and safety, occupational safety and health, sensor devices, robotic devices, machine learning algorithms, future of work

## Abstract

Introduction: The field of artificial intelligence (AI) is rapidly expanding, with many applications seen routinely in health care, industry, and education, and increasingly in workplaces. Although there is growing evidence of applications of AI in workplaces across all industries to simplify and/or automate tasks there is a limited understanding of the role that AI contributes in addressing occupational safety and health (OSH) concerns. Methods: This paper introduces a new framework called Risk Evolution, Detection, Evaluation, and Control of Accidents (REDECA) that highlights the role that AI plays in the anticipation and control of exposure risks in a worker’s immediate environment. Two hundred and sixty AI papers across five sectors (oil and gas, mining, transportation, construction, and agriculture) were reviewed using the REDECA framework to highlight current applications and gaps in OSH and AI fields. Results: The REDECA framework highlighted the unique attributes and research focus of each of the five industrial sectors. The majority of evidence of AI in OSH research within the oil/gas and transportation sectors focused on the development of sensors to detect hazardous situations. In construction the focus was on the use of sensors to detect incidents. The research in the agriculture sector focused on sensors and actuators that removed workers from hazardous conditions. Application of the REDECA framework highlighted AI/OSH strengths and opportunities in various industries and potential areas for collaboration. Conclusions: As AI applications across industries continue to increase, further exploration of the benefits and challenges of AI applications in OSH is needed to optimally protect worker health, safety and well-being.

## 1. Introduction

**Figure 1:**
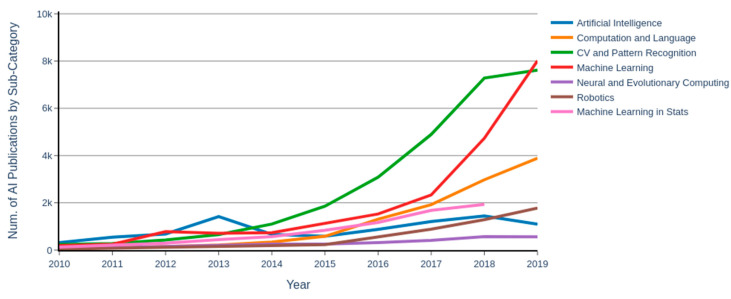
The number of AI papers on ArXiv over time by subcategory from 2010 to 2019. The *x*-axis of the graph is the year of publication (collected from 2010 to 2019) and the *y*-axis is the number of the AI papers on ArXiv split by sub-categories of AI research. The data is provided by “2019 AI Index Reports by Stanford” [1].

**Figure 2:**
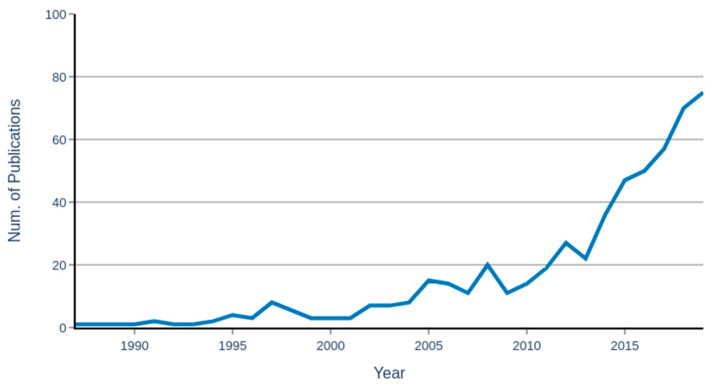
The number of publications demonstrating the use of AI in the OSH field from 1986 to 2019. The *x*-axis is the year of publication and the *y*-axis is the number of the AI papers published with an OSH application. All AI papers queried were individually reviewed to confirm the OSH application.

Artificial intelligence (AI) is an extensive and diverse research field that has infiltrated every aspect of our lives and gained decisive importance over the years with over 20,000 publications in 2019 alone (Figure 1) [1]. In basic terms, AI is the ability of a computer to process information and generate outcomes that mimic how a human learns, makes decisions, and solves problems [2]. While research in AI is relatively new, the concept of AI can be traced back to as early as the 1940s where Alan Turing was one of the first mathematicians to explore the mathematical possibility of AI by posing “whether a machine can think like a human or not” [3]. The term “artificial intelligence” was proposed in a series of workshops at the Dartmouth Summer Research Project on Artificial Intelligence (DSRPAI) hosted by John McCarthy and Marvin Minsky in 1956 [4]. Academia and industry have applied AI to solve various problems such as decision making [5], environmental monitoring [6,7], lower operational costs [8], and increase productivity [9]. The advent of technological advances in robotics, sensors, data management, and computer technology on one hand, and powerful machine learning (ML) algorithms, on the other hand, have opened vast opportunities to apply AI in various fields (Figure 2). For example, ML algorithms are being used to: optimize the performance of a network of sensors used for detecting moving objects [10], select the location of radio frequency sensors used by police/firemen to detect indoor crews in the event of a fire or other threats [11], detect vocal disorder among workers who use their voice maneuvers extensively such as singers and teachers [12], and used to predict bankruptcy [13]. Other important AI applications include: facial recognition technology for law enforcement [14], improvement in marketing and customer service [15], and dramatic improvements in the accuracy of digital imaging [16,17]. These studies point to accumulating evidence that AI technology could effectively be used to detect, identify, and predict risky behavior in a potentially hazardous working environment.

### 1.1. Basics of AI and ML

**Figure 3:**
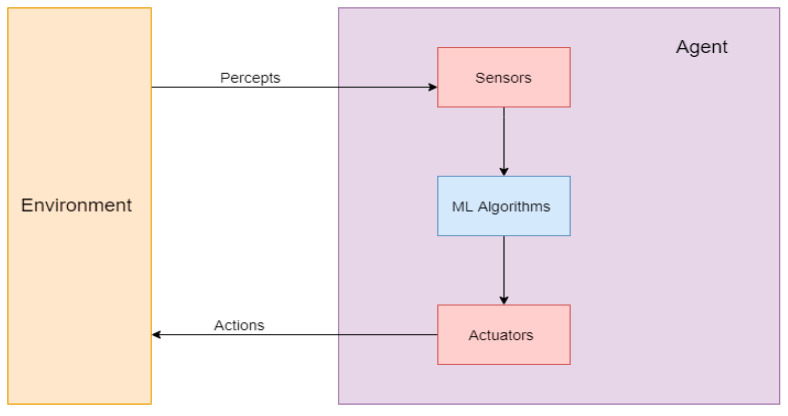
Components of an AI system. Data from the environment is inputted into the AI agent through sensors. This data is transformed/analyzed by ML algorithms that then instruct actuators to conduct certain actions on the environment.

One of the definitions of AI is the study of an agent that receives data from the environment, analyzes the data, and performs an action based on the analysis [18,19,20,21]. The process could be initialized by a collection of the data from the environment by sensor devices, followed by analyzing the data through ML algorithms, and finally, performing the action by actuators (Figure 3). Sensor devices and actuators are considered as the autonomous part of the AI, while ML techniques are the algorithmic part of the AI. In general, ML is considered a sub-division of AI that provides the system with the ability to learn and improve from experiences automatically [21]. In other words, ML is a wide range of algorithms that build a mathematical model based on sample data or features to make predictions or decisions without being explicitly programmed to perform the task [22]. The ML algorithms are capable of learning by trial and error and improving their performance over time [2]. Throughout the paper, the usefulness of both the autonomous and algorithmic applications of AI in OSH for several industry sectors are presented. Since ML techniques play a crucial role in this process, it is essential to understand the algorithms associated with each application.

#### 1.1.1. Types of ML

The purpose of ML models is to make predictions, obtain cognitive insights, and support decision-making [2]. ML uses an advanced set of rules called algorithms to build models. ML algorithms can be divided into supervised learning, unsupervised learning, semi-supervised learning, reinforcement learning [23]. Table 1 shows the type of ML techniques as well as common algorithms for each of them. Each of these algorithms can be defined as follows:

- Supervised algorithms use labeled data that have been previously validated to train a model. The trained model is used to find patterns and make predictions on the unlabeled test dataset [24]. Supervised learning can be divided into regression and classification subsections. Regression predicts a single output value based on different continuous target variables input, for example, prediction of house prices based on different variables such as the location, and size of the house. On the other hand, classification organizes the outputs based on some categorical input variables, for example, predicting if a person is the defaulter of a loan or not.
- Unsupervised learning does not use labeled data for training. Unlabeled data are provided to the learning algorithm, and the model then describes the hidden structure of the data without human guidance, separating the data into clusters or classes [25]. An example of unsupervised learning is that the customers of any specific store or a company such as Amazon can be grouped into different categories based on their similarities in their purchasing histories.
- Semi-supervised learning algorithms analyze unlabeled data mixed with a small number of labeled data. By combining labeled and unlabeled data together, the accuracy of the ML models is improved, and it significantly reduces the costs of prediction of unlabeled test datasets through using supervised algorithms [26].
- Reinforcement learning is a form of sequential learning where the machine is generating its own training data through interaction with a dynamic external environment and optimizing the outcome [27]. Reinforcement models learn the correct outcomes through rewards and penalties using trial and error methods used by humans [2]. For example, if we have an agent, a reward, and many hurdles in between them, the agent will try to find all possible paths to reach the reward. After that, the agent chooses the path with the least number of hurdles to reach the reward [28].

A major class of ML algorithms are constructed based on Neural Networks (NN). NN are designed based on the human brain with interconnected neurons. The NN mathematically adjust the probability weights between nodes in a layer which is called a hidden layer so that the difference between the input and output layers narrows until the actual output of the network matches the desired output [29,30]. Moreover, Deep Learning (DL) is a subdivision of ML that uses a neural network with multiple processing hidden layers of interconnected neurons between input and output layers to recognize a pattern. Convolutional Neural Networks (CNN), and Recurrent Neural Networks (RNN) are popular NN that are used for the implementation of DL algorithms [31]. 

#### 1.1.2. Types of Metrics to Evaluate ML Models

ML algorithms can be evaluated with different metrics, which are given in Table 2. ML models can produce true positive, false positive, true negative, and false negative outcomes. True positive (TP) is an outcome where the model correctly predicts the positive class. False-positive (FP) is an outcome where the model incorrectly predicts the positive class. True negative (TN) is an outcome where the model correctly predicts the negative class. False-negative (FN) outcome where the model incorrectly predicts the negative class [31]. The results of the findings are used to calculate precision, specificity, sensitivity, accuracy, F_1_ measure, and receiver operative character (ROC), in which the formulas for calculating these metrics are presented in Table 2. Precision is the proportion of positive prediction that was correct. Specificity is defined as the proportion of actual negatives, which got predicted as the negative (or true negative). Sensitivity is a measure of the percentage of actual positive cases that got predicted as positive (or true positive). Sensitivity is also termed as recall. Accuracy is the fraction of predictions the model got right among all outcomes. F_1_-measure combines precision and sensitivity values. In the end, Sensitivity and Precision measures are used to plot the ROC curve.

#### 1.1.3. Statistical Modeling versus ML Modeling

ML techniques are built upon statistical frameworks, but they are different from the traditional statistical modeling techniques, such as linear and logistic regressions. In statistical analysis, modeling is dependent on the distribution of the data, but in an ML approach, the development of a model is independent of the distribution of the data. Moreover, in statistical analysis, a model is mostly developed based on all the available data, however, in both the ML supervised and unsupervised learning techniques, a model is developed on a training dataset and will be evaluated using a separate dataset called the testing dataset. The decisions which can be made by statistical analysis are usually involved in a few decision steps; but by ML methods, complex decisions more similar to that of the human brain can be made. Statistical methods use the collected data to infer the relationships between variables, while ML obtains a general understanding of the data to make predictions [32]. The analysis of big datasets using standard statistical analysis is challenging, especially when the number of measurements exceeds the number of individuals and may be further complicated by missing data for some subjects and variables that are highly correlated [33]. ML techniques are especially very advantageous when dealing with such big data sets.

#### 1.1.4. Available Datasets

Datasets are an integral part of the field of machine learning; they are essential as they provide us with the information used to construct the ML models. There are many freely available datasets in a variety of fields such as health care, education, and manufacturing which have been used as the inputs of the ML algorithms. An example of a primary freely available health care database is Medical Information Mart for Intensive Care (MIMIC). MIMIC-III is a clinical dataset owned by the MIT Lab for Computational Physiology, containing de-identified health data associated with intensive care unit admissions including demographics, vital signs, laboratory tests, medications, etc. [34]. Colombian Institute for the Evaluation of Education (ICFES) is a popular educational database with a variety of economic, social, and academic attributes of the students [35]. These attributes have been widely used to develop ML models to predict the performance of the students throughout the semester. The Manufacturing Execution System (MES) is a database that has provided real-time data for various manufacturing activities [36]. These data have been used to create ML methods to optimize production, process quality, and productivity. Another freely available example of databases is Kaggle. Kaggle is an open repository of published data and codes in a variety of fields [37]. These codes and datasets repositories allow users to make their own ML models as well as collaborate with other researchers to build even more powerful models and improve the results. However, no specialized datasets for occupational safety and health (OSH) are available.

### 1.2. AI and OSH

The field of OSH is a subdivision of public health science and integrates disciplines such as toxicology, epidemiology, and ergonomics to study the distribution of illnesses and injuries in the workplace and implement strategies and regulations to prevent them [18]. There has been increasing interest in integrating AI research within the frameworks of OSH. The National Institute for Occupational Safety and Health (NIOSH) founded the Center for Occupational Robotics Research (CORR) to assess the impact of robotics and artificial intelligence on worker safety, health, and well-being in the work environment [19]. Similarly, the European agency for health and safety has also studied the use of AI-enhanced tools and applications in workplaces, looking at where they function and how this is occurring and what the implications are for workers’ occupational safety and health [20]. The number of AI publications with OSH topics has dramatically increased and including several review papers on various industries (Figure 3). Several publications have reviewed the application of AI in OSH in various industries. However, they are limited in scope, and they don’t provide an overall perspective on AI applications in OSH. As the number of AI applications in the workplace dramatically increases, there is a crucial need to gain a thorough understanding of AI methods and their potential impact on worker health and safety.

The objectives of this paper are: (1) define and apply a novel framework to evaluate AI literature in OSH; (2) identify research studies that highlight current applications of AI to improve the health and safety of workers in agricultural, oil and gas, mining, transportation and construction industries; and (3) describe, across these industries, the potential applications of AI in anticipating and controlling occupational hazards, and opportunities for future AI interventions.

## 2. Materials and Methods

### 2.1. Risk Evolution, Detection, Evaluation, and Control of Accidents (REDECA) Framework

**Figure 4:**
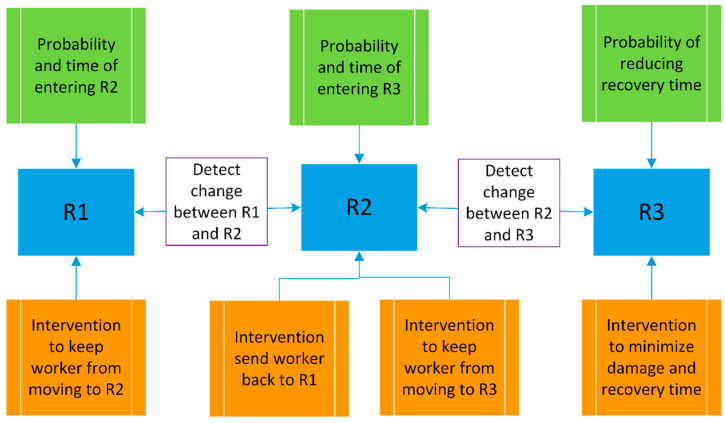
The Risk Evolution, Detection, Evaluation, and Control of Accidents (REDECA) framework for AI OSH. Blue boxes are the different states a worker can find themselves in. R1 is the state when a worker has minimal to no risk of exposure. R2 indicates exposure to hazard and an increased risk of injury. R3 indicates a harmful work-related event occurred. Green boxes indicate technologies that can predict the probability of transitioning into the next states. White boxes are technologies that can detect transitions between the states. Orange boxes indicate the intervention strategies to keep the worker safe or reduce the impact of a work-related event.

Figure 4 describes a novel framework called Risk Evolution, Detection, Evaluation, and Control of Accidents (REDECA) developed by the authors to theorize how AI technologies and methods can be used to anticipate and control risk of exposure in a worker’s immediate environment. The REDECA framework is constructed based on the fundamental underlying idea of the Swiss cheese model [38] that is a dominant paradigm for depicting how injury incidents in complex systems occur. Based on this model a given hazard can generate a safety incident when multiple layers of defenses and safeguards (or interventions) designed to prevent the incident or loss fail to properly act. While the Swiss cheese model conceptualizes that a safety incident occurs when multiple stages of safeguards fail, it is not capable of showing how AI can be used in each step of this process. To create this capability, we extend the Swiss cheese model by including new details that are necessary to describe how AI can be and has been used in detecting, preventing, and controlling the evolution of safety accidents. These details include the characteristics of each state visited when reaching from a safe state to an accident state, the probabilities and timing information associated to each state, and the interventions that can reverse or slow down such a process. We have shown all these details by the REDECA framework shown in Figure 4. We assume that a human worker, due to the nature of his work, can be at different levels of safety risk at any given time. These levels are shown by the three states of R1, R2, and R3 (shown by blue boxes in Figure 4). R1 is the ideal state where a worker has minimal to no risk of exposure to the hazard. Our goal as OSH professionals is to keep the worker in this state. However, this is often not achievable due to the work requirements, available technologies, environmental factors, budget, etc. In R2 the worker is at an increased risk of a harmful work-related exposure event but has not experienced a harmful event. R3 is the state when a harmful work-related event has already occurred impacting the health and safety of the worker. AI technology- based inputs can monitor and foresee the change in state of risk and impact movement between these states of risk to minimize damage from a harmful work-related event. To minimize the chance and negative consequences of safety incidents, we are interested in three types of information and actions related to the states R1, R2, and R3: 1—transition probabilities and times for moving from a lower risk states to a higher risk state (green boxes in Figure 4), 2—detection of a state change (white boxes in Figure 4), and 3—interventions in each state that reduce the risk level or negative consequences of safety incidents (orange boxes in Figure 4).

For a worker who is in state R1, we are interested in AI based models and technologies that help us with the followings: calculating the probability and/or time left for the worker to transition from the safe state (R1) to the hazard-exposed state of R2; detecting (sensing) the event that shows such a transition; and designing and implementing AI based technologies that keep the worker in R1 or at least reduce the probability of moving from R1 to R2.

For a worker in state R2, we are interested in the AI models/technologies that assist us with the followings: calculating the probability and time left for a worker transition from the hazard-exposed state of R2 to R1 or R3; detecting or sensing the events corresponding to these transitions; and the design and implementation of AI technologies and models that could send the worker back to the R1 state or at least reduce the probability of having a safety incident, i.e., moving from R2 to R3.

If a worker experiences an injury incident, then the worker’s state is set to R3. In this state, we are interested in AI models/technologies that help in reducing the damage and recovery time of the worker, and in calculating the times and probability of recovery.

All the AI/OSH papers reviewed by the authors are related to at least one of the green, white or orange boxes shown in Figure 4. Therefore, we use this framework to classify AI/OSH literature related worker’s safety in the five industries of agricultural, oil and gas, mining, transportation and construction.

### 2.2. Literature Search Strategy

**Figure 5:**
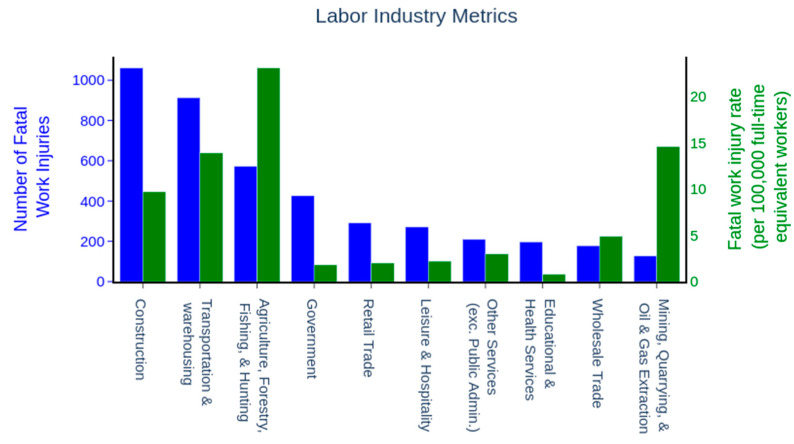
U.S. Bureau of Labor Statistics 2019 survey on the rate of fatal work injury by industry sector [39]. The *x*-axis is the fatal work injury rate and the *y*-axis is different industries.

The five most dangerous industries by fatal injuries are agricultural, mining, oil and gas, transportation, and construction respectively [39]. In 2019, according to the U.S. Bureau of Labor Statistics these industries experienced almost 2700 fatal injuries, 50% of all fatal injuries reported that year. These industries also had over 204,000 injuries that resulted in days away from work, approximately 25% of all injuries in 2019. Moreover, these industries had the highest fatal injury rates of all other industrial sectors and were chosen for this review paper (Figure 5) [39]. The application of AI and ML algorithms, actuators and sensors in the OSH field for these industries were reviewed by using PubMed, Google Scholar, and Scopus search engines to find relevant research. Different keywords such as “artificial intelligence”, “occupational safety and health”, “agriculture”, “mining”, “oil and gas”, “construction”, “transportation”, “ergonomic”, “risk factors”, “sensors devices”, “robots” and their combinations were used to explore available papers in the fields of AI and OSH. For each selected paper, a backward and forward citation search was conducted to capture additional papers not found in the original queries. Over 650 abstracts were reviewed and only papers that were non-repetitive, English-based, relevant to OSH, AI and the five industrial sectors were chosen for further review. The full text of the remaining publications was then read and only papers that meet the criteria specified in our REDECA framework and within the five industries (agriculture, mining, oil and gas, transportation, and construction) were included in this paper.

Each paper was reviewed and classified using the REDECA framework (Figure 4) and AI system (Figure 3). The algorithms, sensors, actuators and environment used/described by the paper were organized by industry in tables highlighting where the majority of AI research in each industry is located within the REDECA framework and AI system. Each component of the REDECA framework was used in the tables using shorthand descriptions and components where there was no available research included in the tables to highlight potential research gaps (Table 3).

## 3. Results

### 3.1. Agriculture

While the agricultural industry has come a long way from its humble origins of subsistence farming to present-day farming, the fatal incident rate has stagnated since the 1990s and it is now considered the most dangerous industry in the US [39]. The number of fatal incidents has dropped from around 1000 cases in the early 1990s to less than 600 cases in 2019. However, the primary attribution of this drop is the reduction in the number of workers (thus less exposure) due to the implementation of more efficient machinery and systems [40]. Precision and digital agriculture research have grown tremendously in recent years due to technological advances in sensor technology, development in robotics human-robot interaction (HRI) [41], unmanned aerial vehicles (UAV) [42], and sophisticated machine learning algorithms. However, significant investments are needed to continue improvements in productivity and enhance health and safety in agricultural environments [43]. The emerging research into collaborative robots (co-robots) is gaining attention as a strategy to create a safe working agriculture environment. The field of human-robot interaction involves designing, developing, and evaluating strategies to help and improve human-robot capabilities and skills together [44]. HRI enabled robots are currently used in urban search and rescue [45].

The agricultural industry is repetitive, labor-intensive, and usually involves lifting heavy loads, which tend to increase the risk of injuries [46,47]. In addition, workers utilize hazardous machinery such as tractors, augers, power take-off (PTO) shafts, grain bins and have to deal with hazardous agents such as pesticides and manure [48,49,50,51,52]. Most agricultural tasks are performed by human-operated machines with some autonomous robots that can work on large-scale fields [53]. The most common injury incident type in the agriculture industry is a collision with a machine or machine parts, which is mainly associated with errors in human factors [54]. Thus, most AI/OSH papers in agriculture reviewed by the authors tended to revolve around HRI strategies that could improve agricultural processes, such as the hazardous tasks of spraying pesticides and the repetitive tasks of the detection of fruits and vegetables, grasping, detaching, and transport procedures [55,56] (Table 4). Three major research areas (robotics, drones, biological sensors) are explored further in this section.

#### 3.1.1. Robotics

For agricultural workers to obtain successful crops, many factors will need to be controlled and monitored, and agricultural robots could be used to perform these repetitive tasks during planting, crop management, and harvesting efficiently and safely while reducing costs.

Yaghoubi et al. summarized a report on the introduction of robotic systems for land preparation [57]. The tasks that robotics systems could optimize include spraying and water irrigation procedures in farms [58,59,60,61], grafting and cutting [62], weeding [63], pruning [5], monitoring and inspection of crops [64,65,66,67,68,69], to map or monitor crop conditions, natural resources, regulating in weather conditions [70,71,72,73,74]. Freitas et al. have shown that a human working with a robot was able to trim trees faster (more than 2×) compared to humans working alone using a ladder-based approach [75]. Similarly, using robots with the relevant sensor utilizing ML algorithms would be able to reduce harvesting workload by handling heavy material and performing repetitive work. Harvest and storing crops at the right conditions via accurate detection and classification of crop quality are explained in the following research [76,77,78]. Bechar et al. were able to show that HRI could be used to improve automatic target recognition of melon on average between 94–100% at a 20% decrease in the time consumed compared to manual operation [55]. HRI collaboration does not need to be a static type. Tkach et al. developed a real-time dynamic switching between collaboration levels in a human-robot target recognition system (the ability to see and recognize what you are seeing). These developments enabled real-time adaptation of the combined human-robot system to make many possible changes in the environment, as well as human operators, and robots initiated the operation. Their ability to correctly recognize what they are seeing was increased by up to 90% [79]. Furthermore, a localization system for HRI robots was developed where vehicle position is triangulated from low-cost wheel encoders and LiDAR sensors without the use of expensive satellite GPS systems [80]. This system allows the robot to track and control its position independently from the operator while spraying or crop detection.

#### 3.1.2. Drones and Remotely Operated Systems

UAVs or drones help in mapping and crop monitoring. Computer vision via sensors and ML algorithms can process data captured from drones flying over their fields [81,82]. Using drones reduces the need for farmworkers to be venturing into remote locations. In high-resolution imagery, shadows may cause problems in the soil and vegetation recognition, Al-Ali et al. used data obtained from a UAV with multispectral sensors to assess vegetation coverage using SVM and maximum likelihood algorithms [83]. The same multispectral camera fixed on UAV technology was used to collect the data, and ML algorithms were used to discriminate between weed and vegetation with an overall accuracy of 96% [84]. The efficiency spraying of pesticides was shown to be improved by using ML algorithms to detect the exact locations reducing the need for workers’ exposure to pesticides [85].

To reduce the physical presence of workers in areas to be sprayed with pesticides, the performance of the robots and ML algorithms needs to be accurate. The following reports have proposed the use of semi-autonomous operation of a teleoperated pesticide-spraying robot [86,87,88,89]. The operator would be able to remotely control the robots using a mouse, a remote, and digital, thus reducing the risk of exposure to hazardous pesticide exposure. For such a strategy to be successful, the following features should be optimized: visibility, safety, simplicity, feedback, extensibility, and cognitive load reduction. Adamides et al. assessed the awareness of the operator and HRI robot, where both should be aware of the status, activities, and the surrounding limitations of the other party [90]. To improve the ambient awareness of agricultural robots, Reina et al. proposed a multisensory perception system by using sensor technologies such as LiDAR, six radar, stereovision, and thermography to detect and avoid obstacles [91]. Berenstein et al. applied two parameters: human-robot collaboration levels and a spraying coverage optimization function (SCOF) in a case study of detecting non-uniform grape clusters in vineyards by allowing both the human and the robot to mark the area for pesticide spraying [92]. Bernstein et al. presented three types of human-robot collaboration: fully manual mode (robot suggests, and human approves), semi-manual (robot sprays and human supervise), or fully autonomous robot spraying modes.

#### 3.1.3. Biological Sensors

Smart robots could also be used to monitor the health of human operators as well. Sensors such as electromyography (EMG) can measure the psychophysical feature of the human operator to modify tasks to improve safety or increase efficiency. For example, Gomez-Gil et al. used EMG readings to steer a tractor with almost the same accuracy as with manual steering [93]. Szczepaniak et al. developed models to assess the stability and steerability of agricultural machines that could be adapted to drivers’ characteristics to improve safety [94]. Sensors were also developed to measure vibrations experienced by farmers using agricultural aircraft. The tri-axial accelerometer sensors were used to measure the acceleration occurring at the level of the seats [95]. Kociolek et al. showed that the operators on quad bikes were exposed to head and neck vibration higher than the permissible level of exposure [96]. Similarly, Calvo et al. used three different accelerometers to measure hand-to-arm vibration and occupational repetitive action (OCRA) level for farmers who always used power tillers and the result indicated the vibration exposure was far above the permissible level of exposure [97].

#### 3.1.4. Summary

A summary of published articles shows that the utility of sensors, robots, and ML algorithms, which are all parts of AI, impact the two main agriculture processes: planting and maintaining the crops, and harvesting; and also human factors. The goals of these technologies are to improve the working conditions in the farms by reducing the need for humans in repetitive and hazardous tasks on farms. For an efficient design and training of sophisticated HRI systems, a detailed study of each task is needed by creating work models from operators to inform technology design and training [56]. Additionally, robotics could be designed to perform more than one task simultaneously to enhance crop and flower production on one hand [98], and harvesting on the other hand leading to improvements in the safety and health of the workers in agricultural environments. In addition to robotics, drones and biological sensors are expected to contribute to the safety and health of farmworkers.

### 3.2. Oil and Gas

The oil and gas industries are defined as any industry directly involved in extracting oil and gas material from the ground and related support activity. The industry uses processes and machinery for the exploration, extraction, refining, transporting, and marketing of petroleum products these days. The oil and gas industries are integrating various advanced sensor technology for collecting data to be analyzed by ML algorithms and to monitor and control the process involved in oil and gas production. The goal of using these technologies is to increase efficiency, reduce costs and at the same time maintain a safe working condition for workers in the oil and gas industry.

The procedure for obtaining oil and gas is divided into three main sectors: upstream, midstream, and downstream. The upstream sector is the exploration of underground and underwater sources of crude oil and natural gas using different apparatus and methods. Once oil or gas is found, it is removed to the surface. The transport of the extracted crude products to the refineries in the midstream sector. The downstream sector involves the refining of crude oil and natural gas and their retail distribution. It is essential to control, monitor, maintain, and secure these processes in every industry and ensure the safety and health of individuals involved in these processes [99].

Advances in ML algorithms, sensors, and robotic technologies to the oil and gas industry have resulted in significant improvements in the safety and health of the workers in their workplaces. A major part of operations in the oil and gas industry may take place in remote locations, hostile and rough terrains where the weather is inclement and harsh. These conditions seem to be worrisome for the safety and health of the workers. Therefore, most AI/OSH papers focused on monitoring, maintaining, and managing industrial operations as well as equipment to detect any potential condition that might be a risk to the safety and health of the workers (Table 5). Smart robots have been used for drilling, inspection, and erosion control in harsh environments in the oil and gas industry which have been useful in improving the safety and health of the workers [8]. Three major research areas (wireless sensor networks, internet of things, machine learning algorithms) are explored further in this section.

#### 3.2.1. Wireless Sensor Networks (WSN)

It is essential to maintain the health and safety of workers during the exploration of oil and natural gas as the increase in temperature or gas levels could indicate a defect in the wells. A WSN was developed by Barani et al., and Ibrahim et al., to remotely monitor the conditions of oil wells using the level, temperature, and gas sensors [100,101]. Aliyu et al., developed a wireless gas safety and monitoring system (WG-SMS), a gas leakage warning system, containing a WSN made up of wireless environmental sensors that are shown to detect toxic and combustible gases accumulating in gas wells. The sensors were solar and battery-powered to reduce energy requirements and were shown to communicate with a command center which would warn of gas leakage and locate workers in danger via GPS [102]. There are several reports of WSN systems used to monitor the different stages of transporting and storing oil and gas in the midstream sector. Disruption of the midstream operations could lead to oil spills and gas leakage with detrimental consequences to the wildlife, environment, and safety of the workers in this industry as well as other humans. Gas leaks occur every year, with many of them leading to injuries, and deaths of humans, equipment damage, and often disastrous environmental effects. The following reports demonstrate the use of real-time data captured from pipeline sensor nodes (PSN) measuring structural stability of the pipelines, oil and gas leakage, and analyses weather and environmental conditions to generate a risk management protocol [103,104,105,106,107,108,109,110,111,112,113,114]. Ding et al., developed a monitoring system to detect pipeline leakage through the negative pressure wave (NPW) features which were collected through the pressure sensors and ZigBee technology [7].

The WSN technology could also be applied to downstream operations of refining crude oil and natural gas and then its retail distribution. Imran et al., used the WSN technology to autonomously monitor and detect any defects in the different downstream operations [115]. Watching the machine conditions to expose any errors was proposed by Hou et al., by using sensor nodes on the machine [116]. Algorithms were developed to analyze data from the sensors for fault classifications. Jung et al., proposed and implemented a WSN based monitoring system for pipe rack safety using data collected from field nodes connected to remote servers by radio frequency transmission modules [117]. Chraim et al., developed and evaluated a wireless gas leak detection and localization solution by using a monitoring network of wireless devices and detection and localization algorithms. A detection rate of 91% is achieved [118].

A WSN pipeline monitoring system was used to detect and localized leakage and blockage in oilfield pipelines [119]. Guo et al., used features from environmental sensors such as wind speed and direction, humidity, and temperature to develop a real-time and large area wireless monitoring system for gas leakage [120]. 

#### 3.2.2. Internet of Things (IoT)-Robotics

Khan et al. describe a new Internet of Things (IoT)-based system to make data collection from connected objects, as simple, secure, robust, reliable, and quick that could be applied to any of the three sectors of the industry [121]. Priyadarshy et al., reviewed in detail the IoT applications in wearable watches, smart helmets, and smart glasses [122]. These devices were used by oil field engineers in offshore fields for real-time assistance, safety, and communication with the control tool for navigation and enhanced collaboration. 

Kim et al., proposed an autonomous sensor-based system named sensor-based pipeline autonomous monitoring and maintenance system (SPAMMS) that combines robot agent-based technologies with sensing technologies for achieving active and corrective monitoring and maintenance of the pipelines [123]. The sensor technology could also be applied to remote terrain such as an underwater system. Felemban et al., surveyed methods for anomalous events in the oil and gas industry, such as detection of pipeline leakage detection with emphasis on software-based methods [124]. 

Some examples of such technologies include digitization of oil fields, real-time optimization of drilling operations, the use of nanotechnology, WSN to aid gauging, reservoir modeling, and diagnostics [125]. Other examples include real-time data collected by sensors are used to ensure better control and optimization of crude production, robots for drilling, inspection, and damage control to enhance efficiency and personal safety, WSN that monitors and improves production, as well as to detect and prevent issues with regards to health and safety. 

#### 3.2.3. Machine Learning Algorithms

There are several reports in the literature demonstrating the contribution of several types of ML methods in the three main sectors of the oil and gas industry. Successful ML algorithms that have been effectively applied in the oil and gas industry include SVM, artificial neural networks (ANNs), and DL which contributed to provide a safer environment for the workers in this industry. There are several reports of machine learning algorithms used in the exploration of oil and gas [126], and drilling [127], reservoir engineering [128], production operations [129], in the oil and gas industry.

The following are some examples of the application of robots with ML algorithms used instead of humans for many tasks that could be risky for the workers and which could facilitate monitoring of leakage, corrosion, or any other damage from remote facilities. Smart robots could intervene to remote areas to assess soil composition during the oil excavation stage [130]. RF and Landsat 8 OLI imagery algorithms were able to map land oil spills [131] efficiently. Jin et al. used LS-SVM to detect leak levels on a gas pipeline based on the acoustic wave method with high accuracy [132]. Robots are used in offshore fields for drilling, inspection, and erosion control to enhance efficiency and personal safety. Mashreq has developed another autonomous robot that has been used for pipelines and other equipment inspections [133].

#### 3.2.4. Summary

As a result, setting overall goals and delegating decision-making to autonomous systems is one of the best things companies can do in such adverse conditions to improve the safety and health of the workers. For example, the introduction of smart robots in drilling and inspection of the various processes and equipment found in the harsh and hazardous environment of the oil and gas industry has improved the safety and health of their workers. These robots reduce worker exposure to extreme temperature, pressure, and humidity. In addition, they are sent into confined spaces, thus reducing exposure to physical and environmental hazards found in these spaces. Moreover, ergonomic-related injuries such as lifting heavy items, bending, working in awkward postures, and repetitive tasks have decreased significantly due to the application of smart robots in these workplaces. Also, WSN technology has allowed workers to remotely monitor operations in hazardous, and inaccessible environments, which prevent them from being exposed to such environments.

### 3.3. Mining

AI and ML algorithms have essential roles to play in the mining industry by increasing the efficiency of mineral exploration and improving workers’ safety and health. Like the oil and gas industry, the mining industry puts the health and safety of miners in jeopardy due to the remote and harsh mining environmental conditions. According to the NIOSH, the fatality rate in the mining industry was reported as 10.4 per 100,000 employees in 2018 [134]. Mining operations could be divided into two stages, exploration and extraction. Mining starts by exploring mineral deposits by collecting data from various remote sites. The next step in the process is drilling and extracting the minerals. The exploration and extraction stages of mining are performed in dangerous and hazardous conditions. Workers tend to be in environments with tight working space, poor lighting, inadequate air supply, and under unstable roofs. In addition, these environments tend to accumulate hazardous waste, poisonous gases, metal and non-metal dust particles, toxic substances, and radioactive materials. These factors tend to make mining operations dangerous and a significant source of adverse health outcomes for workers [135]. AI and ML algorithms can be applied to develop autonomous drills that can locate the potential sites identified in the prospecting stage and perform drilling activities [136].

Most of the reviewed papers focused on structural and environmental mine conditions could be used to analyze the main routes that contain hazardous situations and eliminate them by decreasing or completely removing workers from those conditions [136] (Table 6). Also, ML techniques such as decision tree, RF, and NN can predict the outcome of mining injuries and days away from work using an injury dataset provided by the Mine Safety and Health Administration [137]. AI technologies supporting the safety and health of mineworkers can be organized in two broad categories, sensors and wearable devices and are explored further in this section.

#### 3.3.1. Sensors

Deployed sensors can be split into three types: worker-based sensors, environmental sensors, and autonomous robots. One of the first worker-based sensor systems was developed by Johnson who used global positioning systems (GPS) to monitor workers’ locations and movements [138]. More recently, Baek et al. utilized blue-tooth-based motion and speed sensors, and communication sensors modules to ensure a connection with a worker in remote locations [139]. In addition, sensors used in the medical field to measure physiological features such as body temperature, heart rate, blood pressure could be utilized to monitor the health of the workers inside mines [140,141,142]. 

Environmental sensors are used to collect data on the conditions surrounding the worker, including humidity, noise, toxic gases, temperature, light, and dust [143]. One crucial system is an autonomous remote monitoring framework of wireless toxic gas sensors that can monitor the levels of toxic gases, and provide warnings to protect the health and safety of the mineworkers [79]. Another example is using sensors to monitor humidity levels to combat bacterial growth and prevent worker exposure to harmful bacteria [143]. Lastly, acoustic and ultrasonic optical fiber sensors can be used to monitor the noise level [144,145,146].

Mobile autonomous robots and IoT technology play an important role in the occupational safety and health of mineworkers as they provide real-time information about the status of the mine and mine workers and allow the mine workers to avoid hazardous areas [147]. Sinha et al. reported the use of IoT based on ZigBee techniques to actively monitor underground mineworkers and provide immediate assistance during an emergency [148]. As hundreds of mine workers are shown to be involved in machinery-related injuries routinely, the IoT would provide a highly valuable intelligent machine monitoring system [149] that could be effectively used in detrimental working conditions. Mishra et al. developed and established a Zigbee-based WSN and extended it to IoT with an IP-enabled gateway [150]. Lastly, Autonomous Support Systems could reduce the need for human involvement in perilous mining operations such as roof support [151].

#### 3.3.2. Wearable Devices

Wearable devices using different types of sensors can be used in a wide variety of conditions such as to track motion and location, measure extreme environments, and record physiological characteristics of workers, etc. These features could collectively be incorporated to develop an efficient ML algorithm that could detect hazardous situations more promptly to improve health and safety conditions in the mines. There are many examples of wearable smart devices that integrate information from the environment, motion levels, location and activity, and exposure to hazardous materials. The common devices are helmets, watches, cameras, and activity trackers, which are all useful to improve the overall health and safety of mineworkers.

##### Helmets and Respirators

Helmets with wireless sensors are vital for the safety of the workers in the mining industry [151]. Several commercial companies have developed smart helmets with sensors to monitor the worker and their environments, such as Smart Helmet clip and Angel helmet. Deloitte and Expert mining solutions have acquired the Smart Helmet Clip wearable device with sensors that enable situational awareness including worker’s location and vital signs and the presence of dangerous gasses in the environment [152]. Angel helmet contains detecting systems of motion, impacts, active and passive location, and the position of the workers, besides other effective communication systems [153]. 

Hazarika developed a safety helmet for coal mine workers, which is equipped with methane and carbon monoxide gas sensor [154]. This sensor detects changes in the gas concentrations, and the data is transmitted to the control room wirelessly. It will alert the workers of unsafe methane or carbon-monoxide gas concentration thus efficiently preventing incidents. In addition, wearable respiratory dust monitors could protect workers from exposure to hazardous substances found in mines such as crystalline silica [146]. 

Helmet-Cam, which is a device to assess the amount of dust around the workers in the mine, has been tested at mineral mines [155]. This technology has several components which are held together as a system through a safety vest. The components consist of a real-time data-logging, a respirable dust monitor which is attached to the worker’s belt or backpack, a video monitor, and a video camera which is attached to the helmet. The captured video and dust data then transfers and analyzes to a software to measure the concentration of respirable silica dust in the air.

Mardonova et al. have developed an integrated system to improve the health, safety, and efficiency of the mineworkers [156]. Mardonova’s an expandable smart device combining a safety vest, eyewear, helmet, and watch. The system uses a mobile software system that coordinates the information captured from the individual sensor.

##### Watches

Smartwatches integrate the functionality of a regular watch with added features such as motion detection, global positioning system (GPS) navigation systems, and fitness/health tracking features [157].

Parate et al. described a smartwatch that can measure smoking activities via sensors that detect specific hand gestures such as smoking and separate them from many irrelevant hand gestures [158]. Being able to delineate between normal and abnormal body movements is essential in allowing the ML algorithm to understand the status of the worker when they need help or assistance. 

##### Smart Eyewear and Cameras

Smart eyewear with display screens is able to send alerts and notifications of workers who are in remote locations [156]. Delabrida et al. describe a wearable device made of a head-up display (HUD) assembled with Google Cardboard API and sensors that can measure the distance to an object and can take measurement of the wearers’ environment [6].

#### 3.3.3. Summary

As a result, ML algorithms, sensors, autonomous technologies, and wearable devices, are being developed to address the crucial needs of the mining industry while monitoring the occupational safety and health handoff of the mine.

### 3.4. Transportation

Transportation networks are vital to the economy and societal development. Driver fatigue-related traffic accidents are one of the main factors affecting the safety of workers in the transportation industry [159,160]. The US National Highway Traffic Safety Administration estimates that in 2017, 91,000 police-reported crashes involved drowsy drivers (https://www.nhtsa.gov/risky-driving/drowsy-driving (accessed on 8 August 2019)). Operating a car involves a coordinated set of actions that require situational awareness and prompt decisions, and impairment of awareness is linked to increased risk of crashes [161]. The majority of transportation related papers focused on fatigue related issues (Table 7). 

Fatigue is a physiological state of mind and body expressed by several signs and has different intensities [162]. Examples of signs of fatigue include yawning [163], slow reaction time [164], eyelid shutting [165], and loose steering grip [160]. One of the standards that all fatigue algorithms are contrasted against is the Karolinska Sleepiness Scale (KSS) [166,167,168]. The KSS is one of the earliest tools used to measure fatigue levels using a self-administered questionnaire [167]. The KSS is considered the gold standard in measuring fatigue, though it is important to note that it is a subjective reporting method that is not real-time and suffers from recall and reporting biases that might be needed to prevent crashes. 

Fatigue could be classified into active, passive, and sleep-related fatigue [169]. There is a connection between fatigue signs such as drivers’ blink duration and driving performance [170,171,172]. It would be too complex to explicitly code every situation to generate predictive crash models using the usual statistical methods. As a result, machine learning algorithms would be more efficient in detecting and predicting driver fatigue by using information from drivers’ characteristics, vehicle characteristics, or both. Driver fatigue detection algorithms can be categorized based on the data collected by the sensors. These algorithms fit into four broad categories: biological, facial, vehicular, and hybrid algorithms. 

#### 3.4.1. Biological Algorithms

Biological algorithms use real-time data collected from the heart, brain, and muscular activity as indicators of the onset of fatigue [167]. Changes in the heart rate (HR) and heart variability (HRV) from electrocardiograms (ECG) non-invasive sensors embedded in steering wheels could be used to detect driver’s fatigue [173]. Changes in photo platysma gram (PPG) readings could also be used to identify fatigue. Li et al. used a PPG sensor on the steering wheel of the vehicle to measure HRV and the SVM algorithm to categorize drivers into fatigued and alert states with a 95% accuracy compared to the gold standard KSS and PERCLOS [174]. Another biological measurement is electro-oculography (EoG), which is the measurement of the cornea-retinal potential difference between the back and the front of the eye, Zhu et al. used an unsupervised machine learning algorithm to detect fatigue [174]. EoG data was obtained from 22 participants with electrodes around their eyes, and response error was shown to increase with fatigue. Since a sensor near the eye could distract the driver, Zhang et al. developed a sensor on the forehead instead [175]. Electroencephalography (EEG) could similarly be used to assess the onset of fatigue in the brain. The EEG signal is divided into five waves which are associated with different levels of drowsiness [176,177,178]:

- Gamma (30–42 Hz)
- Beta (13–30 Hz): a measure of alertness and early sleep stage.
- Alpha (8–13 Hz): associated with relaxed status
- Theta (4–8 Hz): associated with deep sleep
- Delta (0.5–4 Hz): related to the early stage of sleep

The sensor could also be used to collect information regarding the level of muscle activity from the surface of the skin using a surface electromyogram (sEMG) [179]. Balasubramanian et al. used sEMG to measure muscle activity changes in the shoulder, neck, back, and wrist found that the power in 15–30 Hz frequency increased with fatigue [180]. Although these changes have a high correlation with fatigue, obtaining EEG and sEMG are both intrusive methods that have limitations in their applicability. 

Multiple biological features could be used to improve the performance of the fatigue detection method. Sun et al. used recruited 30 participants to use wearable ECG and EEG sensors and had the EoG sensors fixed on the vehicle ceiling to test his fatigue-detection method [181]. 

Sun et al. observed that the following features were associated with fatigue: (1) increased blink duration and frequency; (2) decreased power density of alpha and beta waves; (3) decreased LF/HF; (4) increased SDNN; increased RMSSD, LF and HF. 

Chai et al., categorized fatigue by developing a feed-forward Bayesian neural network [182]. Their classification system used independent components by entropy rate bound minimization analysis (ERBM-ICA) for the source separation. Also, the features were extracted through an autoregressive (AR) method. At the end, the Bayesian neural network was developed to classify fatigue state versus alert state. The model was evaluated through sensitivity, specificity, and accuracy metrics which are of 89.7%, 86.8%, and 88.2% respectively. In another study, a CNN model was developed to detect fatigue from the EEG signals by Yang [183]. 

#### 3.4.2. Facial Algorithms

Facial expressions, such as eye, mouth, and head movements are the most visible signs of fatigue, and several commercial companies have developed fatigue detection systems relying on drivers features such as yawning, blink duration, and frequency, percent of the time the eyes are closed (PERCLOS), head movement [184]. Applied Science Laboratories (ASL) designed an eye-tracking system using a computer vision techniques system to measure eye movement association with fatigue [185].

In another study, blinking frequency, eye-closed duration, mean of eye-opened level, and yawing frequency were used as the physical features. Moreover, the percentage of non-steering, the standard deviation of steering-angle, frequency of abnormal land deviation, and standard deviation of vehicle speed were used as the vehicle features [186]. Sigari et al. demonstrated that the face tracking method using a fuzzy classifier (fatigue levels: low, normal, and high) was not optimal and complex when testing it on 5 participants in a real driving environment [187].

Mandal (developed a vision-based fatigue detection system for bus drivers by monitoring and testing on 23 bus drivers in a real driving condition [188]. The system consisted of various modules of head-shoulder detection, face detection, eye detection, eye openness estimation, fusion, PERCLOS estimation.

Mouth movement data were also used to predict fatigue. Alioua et al. developed a non-intrusive fatigue detection system by monitoring yawning features with face extraction-based SVM and a mouth detection approach based on circular Hough transform (CHT) [189]. This system was able to detect fatigue with a 98% accuracy when more features were included, but fatigue was simulated, and the number of participants was not reported. 

A different way to approach this problem is to allow deep learning algorithms to learn the features. Dwivedi et al. developed a DL algorithm to select the visual elements for fatigue classification [190].

In another study, the drowsiness of the drivers was monitored through eye movement of the driver, CNN was developed as the ML algorithm to do the prediction of if the driver is drowsy or not, and if he is able to drive safely or not, and the sound alarm was produced when the driver was drowsy [191]. Also, a complex network (CN)-based broad learning was developed to detect fatigue from the EEG signals [192]. 

Many commercial companies have developed facial algorithms to detect fatigue. Smart Eye AB has designed Anti Sleep, a system that uses 3D head position, head orientation, gaze direction, and eyelid closures to detect driver fatigue [193]. The OPTALERT developed by Sleep Diagnostics Pvt, uses wireless glasses to record eyelid and pupil activity which were used as an early warning to inform drivers about fatigue [194]. Driver Fatigue Monitor System MR688, developed by Care Drive alerts the drivers in which detection of features of fatigue or distraction is done by infrared image sensors that record the pupil and head movement [195]. Op Guard is a real-time fatigue and distraction detection system developed by Guard Vent using sensors to measure eye, head, and face movements, and driver’s behavior. It sends immediate notification of fatigue to the driver by remote personnel monitoring the drivers’ performance [196].

#### 3.4.3. Vehicular Algorithms

Vehicle and steering wheel movement patterns can also be used to detect fatigue. Toyota has developed the Toyota Sense P fatigue-detection system by collecting information about the vehicle’s surroundings, lane deviation, and detection of pedestrians [197]. Nissan Maxima tracks the driver’s steering patterns, and if it detects any unusual deviation from the designed model, a warning signal is generated to alert the driver [198]. Volkswagen also offers a similar lane tracking system, pedal use, and erratic steering wheel movements to judge driver fatigue levels [199].

Fatigue reduces influences the driver’s performance that could be captured from driving features such as steering wheel angle, lane deviation, load center position (LCP), and posture changes. 

Measuring steering angle could be used to identify driver fatigue. McDonald et al., developed a fatigue-detection method using features such as lane departures from steering wheel angle data (from modified observer rating of drowsiness) and a RF algorithm. Using 72 participants, the RF algorithm had higher accuracy and ROC than PERCLOS and had a comparable positive predictive value [200]. 

Li et al. used approximate entropy (ApEn) features which were collected under real driving condition from the sensors mounted on the steering lever to monitor the level of driver fatigue and the drowsiness of the drivers [201]. The data included 14.68 h of driving on a Chinese highway, and the model yielded an accuracy of 78.01%. Li et al. illustrate that ApEn features are useful, but further development is needed to improve accuracy. 

Yang et al. recruited 12 subjects to measure a type of different sleep levels (partial versus no sleep sleep-deprivation) test under a simulated driving environment [202]. Several stimulus-response tasks as well as routine driving tasks were performed to analyze the performance differences of drivers under various sleep-deprivation levels. They also demonstrated that sleep deprivation affected rule-based than skill-based cognitive functions where sleep-deprived drivers had power response to unexpected disturbances degraded but were able to continue the routine driving tasks such as lane tracking, vehicle following, and lane changing. Another feature used in fatigue detection is the LPC measured by pressure sensors placed in the seats. Furugori et al., showed that in a vehicle of 12 subjects, the LPC from body pressure sensors was at the beginning distributed throughout the seat, but as time progressed, the pressure started to concentrate more or less at one point toward the back of the seat [203].

Wakita et al. developed a GMM and the Helly models to identify driver fatigue. Multiple features such as vehicle velocity, brake pedal, accelerator pedal, and distance from car in-front were utilized to feed to the models [204]. The GMM model yielded better results compared to the Helly model in terms of accuracy metric which were 81% on a simulator and 73% on a real vehicle.

#### 3.4.4. Hybrid Sensors

Integrating driver data features and vehicular features to detect fatigue could drastically increase the accuracy of fatigue detection methods compared to a single feature (from either the driver or the vehicle) approaches. Cheng et al. proposed a technique that incorporates both driver characteristics as well as vehicle characteristics such as eyelid closure, maximum close duration, and percentage of non-steering percentages, percentages of on-center driving, the standard deviation of lane position [205]. A data fusion framework was developed to model the data from the driver and the vehicle. To measure the feature-level fusion, Fisher’s linear discriminant was used. Also, the Dempster Shafer evidence theory was used in the decision level fusion process. In this study, the vehicle dependent, and the driver dependent measures, were 81.9% and 86.9% accurate respectively. However, the fusion of both measures were more accurate which was 90.7%.

Sun et al. developed a self-adaptive dynamic recognition model. The features were collected from several sources. Also, the sequential levels of fusion were built at both feature and the decision levels [206].

Naurois et al. developed a detection and prediction model using several physiological and behavioral features, recorded driving behavior, driving time, and participant information [207]. These features were then fed to the ANN model to detect fatigue. The best result was obtained once physical features, driving time, and participant information were employed.

Combinations of biological, and vehicular features have been shown to improve the accuracy of the predictive algorithm [208,209,210]. A mobile-based support vector machine (M-SVM) was able to classify the driver state with a 95.8% accuracy. Samiee et al. combined features such as eye status, lateral position, SWA, ECG, EEG, and sEMG from 12 subjects to have a prediction system with 94.63% accuracy [210].

In another work, a dynamic probability assignment (BPA) was introduced to the decision-level fusion. In this approach, the weight of each feature changes dynamically, and the combination of the previous fatigue state and the current step in the decision-level fusion are used to improve the result of the fatigue driving detection. Using the fusion of all fatigue features offers an accuracy of 92.1% (an improvement of 90.8% for vehicle features and 91.6% for facial features) and using only the most useful features offers an accuracy of 93.8% [205].

As a result, due to the complexity of coding every situation to generate predictive crash models using the usual statistical methods, driver fatigue detection algorithms have been developed by using information from drivers’ characteristics, vehicle characteristics, or both to detect and predict driver fatigue more efficiently.

### 3.5. Construction

The construction industry is described as a loosely coupled system with several issues in a typical project [211]. One of these issues is the safety and health of workers at a construction site. According to OSHA data, out of 4779 worker fatalities in private industry in 2018, 1008 or 21.1% were in construction, and among construction fatalities, the leading cause of fatality was falls [212]. Not surprisingly, the focus of most of the AI/OSH papers was on detecting and predicting falls in construction sites (Table 8). Several types of sensors are used to collect data, and a variety of machine learning algorithms are used to analyze and detect falls that are explored further in this section.

#### 3.5.1. Fall Detection Sensors

Fall detection starts with the gathering of data from sensors. It is essential to have a precise measurement of a fall event to develop an efficient fall-detection algorithm. Falls are unexpected, rare, and diverse events. A fall is defined as “an event which results in a person coming to rest unintendedly on the ground or other lower level, not as a result of a major intrinsic event (such as stroke) or overwhelming hazard” [213,214]. There are no typical methods for fall detection in terms of type of sensors, features to extract, and specific ML algorithms to achieve better results. Fall detection has been detected through building information modeling (BIM) technology in several studies [215,216,217,218,219,220,221,222]. Other reports of fall detection methods in the literature could be divided into three categories: wearable devices, ambiance sensor-based, and camera-based [214,223]. Detection of fall helps to get first responders at the scene quickly and potentially reduce the negative health outcomes related to the fall.

##### Wearable Devices

**Figure 6:**
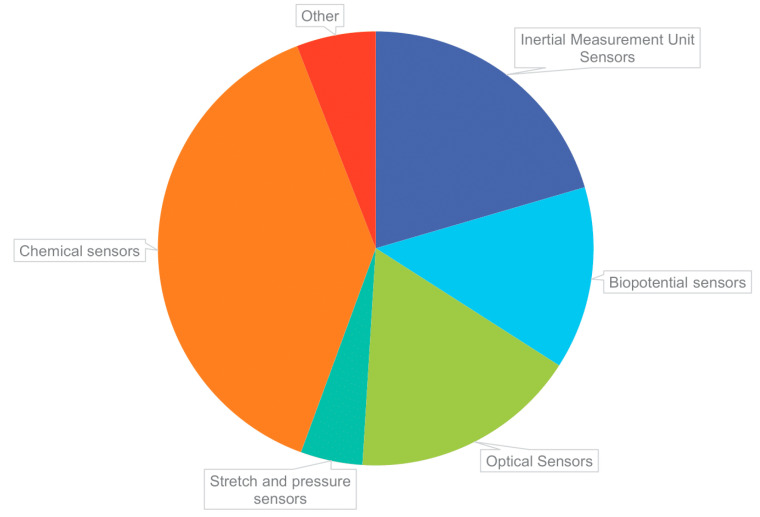
2020 revenue from wearable sensors, the graph is provided by the market research report ‘Wearable Sensors 2021–2031’ IDTeachEx [239]. Copyright © 2021 IDTechEx Research. Some rights reserved.

Advances in sensor technology have led to the development of non-intrusive small, low-cost sensors that could be integrated into devices such as watches or phones, that can monitor workers in real-time. The number of wearable sensors and their market size has risen significantly and is anticipated to be around 3 billion wearable sensors by the year 2025 (Figure 6) [224]. One main reason that the market is achieving prominence is because of the rising number of health and fitness monitoring applications globally such as the health of the workers in the workplaces. 

The most popular features extracted from these sensors are the magnitude of the accelerometer called the signal magnitude vector (SMV) [225,226,227,228,229,230,231,232,233,234,235], the angular magnitude of the gyroscope [232,233], and electromyography [236]. Robust fall detection methods were developed based on acceleration features with sensors placed in the right position in the body [237,238].

##### Camera-Based

A camera-based sensor is non-intrusive equipment for monitoring the user’s environment and behavior by measuring the ratio of the width to the height of an image. Low-cost cameras can detect changes in body movement [238,239,240,241] shape, posture, and head movement.

##### Ambiance Sensors

An ambient device collects information about the changes in the environment surrounding the individual. An ambient sensor such as vibration patterns [242,243], pressure sensors [244,245], detect changes in infrared patterns [246], an electric field [247] which can be used for accurate measurements of various mobility and gait parameters used critically in fall-detection systems [248]. An acoustic sensor makes use of a microphone sensor to capture the movements of the users where Mel Frequency Cepstral Coefficient (MFCC) features are extracted to be analyzed and detect the falls.

### 3.6. Developing Fall-Detection Algorithms

In almost all of the current research efforts to develop a fall-detection model, the following steps are used: data collection from different sensors, extracting the relevant features from the data, developing the classification models and prediction stage, and finally, evaluating the fall-detection system [31]. Selecting the relevant features improves the accuracy of the prediction method by reducing the size of the noise in the dataset [249]. The developed ML algorithm and the types of sensors that are used can affect the accuracy values significantly. Mubashir et al. found vision-based devices are more robust for detecting falls [250]. Yu et al. advocated for generic fall detection algorithms and the fusion of different sensors such as wearable and vision sensors to provide more accurate fall detection models [223]. 

Ojetola et al. were able to discriminate between falls and other similar activities by developing a decision tree model, which resulted in a precision value of 81% and a recall value of 92% [251]. Noury et al. [252] and Yu et al. [223] summarized the systems, algorithms, and sensors used for the automatic early detection of the fall and illustrated the difficulty in comparing the performances of the different methods due to the lack of a common framework [252]. The unsupervised learning methods in the research failed to identify the first fall as it was not observed, and the supervised methods were likely to misidentify regular movements as falls [252]. 

Detection of near-miss fall is also essential as it could evolve into fall incidents in construction sites [253]. Zhang et al. demonstrated the use of smartphones to capture near-miss falls and identify them with an ANN algorithm yielding a precision of 90.02%, recall of 90.93%, and F_1_ score of 90.42%. 

In case of an injury incident on a construction site where the location of workers is needed for rescue efforts, Liu et al. developed position estimation algorithms based on the strength of the radio signals received from multiple wireless access points inside buildings [11].

In addition, machine learning algorithms could be used to detect and predict falls from scaffolding structures. Sakhakarmi et al. designed a method of classifying scaffolding failure cases and reliably predicting safety conditions based on strain data sets obtained from scaffolding columns [254].

Lee et al. developed a sound recognition system. This technology was able to send immediate notification and alarm to the workers when an incident took place. Also, it was able to provide information regarding safety measures that workers should take in the case of unsafe situations before they start their work activities. The sound features were then fed to a ML algorithm to detect the falls [255].

ML algorithms developed by Yang et al. were able to predict potential fall incidents using data of workers’ abnormal gait patterns in a construction site [256]. They found the following four gait parameters (i.e., stride time, stride distance, average velocity, and maximum foot clearance) were better at distinguishing hazardous environments.

As a result, various wearable devices, camera based and ambiance sensors, as well as ML algorithms have been developed to detect the falls.

## 4. Discussion

The application of AI in the realm of several industries has been described as the Fourth Industrial Revolution [257]. Innovations in artificial intelligence through the use of sensors, robots, ML algorithms have been shown to increase productivity and could potentially improve the safety and health of workers in the workplace. Since the application of AI in workplaces has increased over the past few years, it is very crucial to have a thorough understanding of AI methods, and the effects of these methods on the workers and workplaces as well. To aid in this understanding, this paper developed a the REDECA framework to categorize and highlight the applications of AI in OSH. This novel approach is a natural by-product of the literature developed. It was created by carefully reviewing the literature and developing large categories where the papers in the literature fell. The available OSH AI literature was compiled in tables by industry and by AI system element to identify the key strengths, weaknesses, and opportunities. Table 4, Table 5, Table 6, Table 7 and Table 8 categorizes the available literature by which element of the AI system each publication’s intervention focused by the targeted AI approach in the REDECA (Figure 4) framework. This categorization clearly and efficiently highlights the strengths, opportunities, and weaknesses of using AI in OSH.

In brief, the construction industry and evaluating driver fatigue in the transportation industry had many AI algorithms identified in the peer-reviewed literature. These algorithms spanned across most elements of the AI REDECA. Conversely, in agriculture, mining, and oil and gas industries there were very few AI algorithms used. Similarly, we see the agriculture and mining industries have many actuators, when other industries did not. In all industries there were many papers published describing the use of sensors and environment descriptors. The ability to be able to quickly view where there are gaps in the literature across the AI system is strength of using this framework. Another strength of the program is to be able to identify which part of the REDECA is missing AI involvement.

By separating the papers in the published literature into their targeted approach to protecting workers using the AI REDECA it becomes clear that most AI interventions target probabilities, detection, and interventions when a worker is in R1. In general, there is a lack of developed and published material describing AI systems aimed at detecting when someone goes from being exposed to a risk environment (R2) to being injured or put in risk state 3. This then precludes one from establishing how long it will take to return to healthy. There is also an opportunity to develop AI models targeting interventions to keep workers from moving to R3 and interventions to minimize the damage from being in R3 and improve recovery time. When protecting workers, it is important to focus efforts on the early stages of intervention with the goal of never having a worker in R3. Unfortunately, this is not always possible and thus the opportunity uncovered by using this framework is to develop AI systems targeted at reducing the probability and increasing the interventions of workers in R3 of the AI REDECA (Figure 4). These elements are crucial to minimize harm in the event of a workplace incidents.

This paper is not a systematic review of the AI literature. Our paper is the first survey of the reach of existing applications of AI in OSH and documents several examples of how AI can enhance the effectiveness of OSH interventions to protect workers in diverse work sectors. The authors acknowledge the limitations of the current paper and recommend several areas for further exploration:

(1) First and foremost, a systematic review of scientific journals, industry reports and other practice journals may provide insights into more applications of AI in OSH beyond the scope of this survey. Additionally, qualitative approaches may be needed to fully understand the dynamics of AI-OSH teams in the field that have not been captured in this survey.
(2) Our survey did not find any educational papers about AI curriculum or training in OSH. A recent paper specifically highlights the need for OSH professionals, practitioners, researchers, employers, and workers should develop a better understanding of worker health, safety and well-being applications of AI [258]. A comprehensive scan of existing AI curricula in academia and training and skills needs among OSH professionals in industry may provide a better understanding of future AI capacity needs for OSH researchers and practitioners. For example, a significant increase in the availability of funding for AI applications in healthcare over the past ten years has led to a shift in the number of students and healthcare professionals with access to AI training and the capacity to implement AI applications.
(3) Currently there is no dedicated funding source for AI research or practice in OSH. The fourth industrial revolution (also known and Industry 4.0) is here and the NIOSH Future of Work Initiative was launched in 2019 to identify novel research solutions, practical approaches, and stakeholder opportunities to collectively address the future of work [259,260]. AI, including deep leaning, neural networks and machine learning, are priority topics and subtopics listed in the guiding framework for NIOSH research and practice-based activities as part of this initiative [259]. We need to advocate for resources to fund research and training of OSH professionals in governmental agencies (NIOSH), academic institutions and industries to fully leverage the capacity of AI to protect the health, safety and well-being of workers.
(4) AI will continue to play a very significant role in the design of future workplaces, work health and worker well-being. It is anticipated that massive innovation in industries driven by AI could potentially lead to the creation of new sectors for growth and jobs, and eliminate several existing jobs. Recently the European Commission proposed new rules and actions aiming to “turn Europe into the global hub for trustworthy Artificial Intelligence (AI)” [260]. The goal is to “coordinate a plan with Member States to ensure the safety and fundamental rights of people and businesses, while strengthening AI uptake, investment and innovation across the EU” [260]. This aspect of AI was not the focus of this paper but the authors recognize the potential of AI use on occupational health equity (biased outcomes). OSH researchers and practitioners need to advocate for a long-term strategy in partnership with government, AI experts and industry for protecting the health, safety and well-being of all workers.

## 5. Conclusions

AI was founded in 1955 as an academic discipline with the idea that a machine can be endowed with tools that can be made precisely to simulate human intelligence. AI will be ubiquitous in the workplace across all industries and can be used to detect, evaluate and predict hazardous events and environments to improve the health and safety of the workers. Application of the REDECA framework has highlighted AI/OSH strengths and opportunities for advances in sensors, robotics, and machine learning algorithms to greatly improve working conditions in the agriculture, oil and gas, mining, transportation and construction sectors. As AI applications across other industries continues to grow, there is a need for collaboration among OSH and industry partners to more systematically explore the benefits and challenges of AI applications in OSH to protect worker health, safety and well-being.

## Figures and Tables

**Table 1:** Type of ML techniques and the algorithms associated with each technique.

Types of ML Techniques	List of Most Common Algorithms
Supervised ML	Support Vector Machine (SVM), Naive-Bayes, K-Nearest Neighbor, Decision Trees, Random Forests (RF), Linear Regression, Logistic Regression, DL
Unsupervised ML	K-Means, Hidden Markov Model (HMM), Principal Component Analysis, Gaussian Model Mixture (GMM), DL
Semi- Supervised ML	Self-Training, Co-Training, Generative methods, Mixture models, Semi-supervised SVM, Graph-based methods
Reinforcement ML	Q-Learning, Temporal Difference, Deep Adversarial Networks

**Table 2:** Evaluation Metrics.

Evaluation Metrics	Formula
Recall or sensitivity	$\frac{\mathrm{TP}}{\mathrm{TP}\;+\;\mathrm{FN}}$
Precision	$\frac{\mathrm{TP}}{\mathrm{TP}\;+\;\mathrm{FP}}$
Specificity	$\frac{\mathrm{TN}}{\mathrm{EP}\;+\;\mathrm{TN}}$
Accuracy	$\frac{\mathrm{TP}\;+\;\mathrm{TN}}{\mathrm{TN}\;+\;\mathrm{TP}\;+\;\mathrm{FP}\;+\;\mathrm{FN}}$
F_1_-measure	$\frac{2\;\times \;precision\;\times \;recall}{precision\;+\;recall}$
ROC	$\sqrt{specificity\;\times \;sensitivity}$

**Table 3:** REDECA components and shorthand notations used in industry Tables 4–8.

Prob. R2	Probability and time of entering R2
Detect R1→R2	Detect change between R1 and R2
Int. R1→R2	Intervention to keep worker from moving to R2
Int. R2→R1	Intervention send worker back to R1
Prob. R3	Probability and time of entering R3
Detect R2→R3	Detect change between R2 and R3
Int. R2→R3	Intervention to keep worker from moving to R3
Prob. Rec.	Probability of reducing recovery time
Int. R3	Intervention to minimize damage and recovery time

**Table 4:** Agricultural AI/OSH algorithm, sensor and actuator research organized by the REDECA framework. Major technologies described by each paper is mentioned and linked to the relevant papers [39,40,41,42,43,44,45,46,47,48,49,50,51,52,53,54,55,56,57,58,59,60,61,62,63,64,65,66,67,68,69,70,71,72,73,74,75,76,77,78,79,80,81,82,83,84,85,86,87,88,89,90,91,92,93,94,95,96,97,98]. Summary of each paper is found in Appendix A.

REDECA Components	AI Algorithms (ML)	Sensors	Actuators	Environment (Type of Hazard)
Prob. R2	Linear Mixed Model: [59] Signal Detection theory (SDT): [79] *SVM:* [84] *Image Processing*: [85]	Laser: [58] Camera: [59,61,84,85] EEG: [94]	Robot: [41,57,60,61] UAV: [84,85] Tractor: [94]	Musculoskeletal Disorders: [41,57,61] Pesticide: [58,59,60,84,85] Machinery: [79,94]
Detect R1→R2		LIDAR: [91] Radar: [91] Camera: [91] Thermography: [91]	Robot: [91]	Machinery: [91]
Int. R1→R2	Linear Mixed Model: [59] Image Processing: [63]	Camera: [59,60,61,63,78,87,88,89,90] Ultrasonic: [62] GPS: [63] Infrared Laser: [78] Pressure [78] EEG: [93]	Robot: [59,60,62,63,78,87,88,89,90] Tractor: [93]	Pesticide: [59,60,87,88,89,90] Planting: [62] Weeding: [63] Harvesting: [78] Vibrations: [93]
Int. R2→R1				
Prob. R3		Accelerometer: [46] Vibrations: [95,96,97]		Musculoskeletal Disorders: [46,95,96,97]
Detect R2→R3				
Int. R2→R3				
Prob. Rec.				
Int. R3				

**Table 5:** Oil and Gas AI/OSH algorithm, sensor and actuator research organized by the REDECA framework. Major technologies described by each paper is mentioned and linked to the relevant papers [99,100,101,102,103,104,105,106,107,108,109,110,111,112,113,114,115,116,117,118,119,120,121,122,123,124,125,126,127,128,129,130,131,132,133]. Summary of each paper is found in Appendix B.

REDECA Component	AI Algorithms (ML)	Sensors	Actuators	Environment (Type of Hazard)
Prob. R2	SVM: [107,109] GMM: [107,109] KNN: [107,109] ANN: [128]	Temperature: [105,106,119] Pressure: [105,106,107,109,119] Transducers: [107] GPS: [112] Acoustics: [119]		Pipeline Leakage: [100,105,106,107,112,119] Gas Pressure: [105,106,119] Flow: [119] Noise: [119] Temperature: [119]
Detect R1→R2	SVM: [107,132] GMM: [107] KNN: [107] Transform: [113] Localization: [118]	Temperature: [105,106,111,120] Pressure: [105,106,107,109,110,111] Transducers: [107] Flow: [108] Acoustic: [110,111,113,132] Gas Sensor: [118,120] Humidity: [120] Windspeed: [120]		Pipeline Leakage: [105,106,107,108,109,110,111,113,132] Gas Pressure: [105,106,132] Noise: [110,111] Fire: [118] Chemical: [118,120]
Int. R1→R2	Localization: [118]	Temperature: [100,101,103] Pressure: [100,101,103] Gas Sensor: [102,118] RFID: [123]	Robot: [123]	Confined Space: [100] Well head: [101] Drilling: [102] Pipeline Leakage: [103,123] Fire: [118] Chemical: [118,123]
Int. R2→R1				
Prob. R3				
Detect R2→R3				
Int. R2→R3				
Prob. Rec.				
Int. R3				

**Table 6:** Mining AI/OSH algorithm, sensor and actuator research organized by the REDECA framework. Major technologies described by each paper is mentioned and linked to the relevant papers [134,135,136,137,138,139,140,141,142,143,144,145,146,147,148,149,150,151,152,153,154,155,156,157,158]. Summary of each paper is found in Appendix C.

REDECA Component	AI Algorithms (ML)	Sensors	Actuators	Environment (Type of Hazard)
Prob. R2			Robot: [147] IoT: [147]	General: [147]
Detect R1→R2		Motion: [139,153] Accelerometer: [139,145] Gyroscope: [145] Magnetometer: [145] GPS: [139] Humidity: [143,150] Sound: [143,145,146] Temperature: [143,145,150] Toxic gases: [143,144,145,150,152,154] Dust: [143,145] Heart Rate: [145] Infrared: [145] Camera: [145,153] Smoke: [145] Silica: [146]	Smartphone: [145] Smartwatch: [145] Smart helmet: [145,152,153,154]	General: [139,145] Fall: [153] Bacteria: [143] Hearing: [143,146] Toxic gases: [143,144,150,152,154] Temperature: [143,150] Silica: [146] Humidity: [150]
Int. R1→R2		Motion: [139,153] Accelerometer: [139] GPS: [139] Camera: [153]	IoT: [149] Smart helmet: [153]	General: [139] Machinery: [149] Fall: [153]
Int. R2→R1		Accelerometer: [145] Gyroscope: [145] Magnetometer: [145] Heart Rate: [145] Infrared: [145] Camera: [145] Sound: [145] Smoke: [145] Gas: [145] Temperature: [145] Dust: [145]	Smartphone: [145] Smartwatch: [145]	General: [145]
Prob. R3		Accelerometer: [145] Gyroscope: [145] Magnetometer: [145] Heart Rate: [145] Infrared: [145] Camera: [145] Sound: [145] Smoke: [145] Gas: [145] Temperature: [145] Dust: [145,155]	Smartphone: [145] Smartwatch: [145] Smart helmet: [155]	General: [145] Fall: [151] Silica: [155]
Detect R2→R3				
Int. R2→R3				
Prob. Rec.	ANN: [137] DT: [137] RF: [137]			General: [137]
Int. R3				

**Table 7:** Transportation AI/OSH algorithm, sensor and actuator research organized by the REDECA framework. Major technologies described by each paper is mentioned and linked to the relevant papers [159,160,161,162,163,164,165,166,167,168,169,170,171,172,173,174,175,176,177,178,179,180,181,182,183,184,185,186,187,188,189,190,191,192,193,194,195,196,197,198,199,200,201,202,203,204,205,206,207,208,209,210]. Summary of each paper is found in Appendix D.

REDECA Component	AI Algorithms (ML)	Sensors	Actuators	Environment (Type of Hazard)
Prob. R2	GMM: [203] Helly model: [203] ANN: [207]	Infrared: [170] Camera: [170,207] EMG: [180] Pressure: [203] Vehicular: [207]		Fatigue: [170,180,203,207]
Detect R1→R2	SVM: [173,174,175,188,209] CNN: [183,190,191,192] Bayesian NN: [178,182,202,208] Digital Signal Processing: [181] Fuzzy NN: [186,187] DL: [189] Binary Decision Classifier: [200,201] ANN: [207] NN: [210]	Infrared: [170,193,210] Camera: [170,186,187,188,189,190,191,192,193,200,205,206,208,210] ECG: [173,181] PPG: [174,208,209] EOG: [175,181] EEG: [178,181,183] Vehicular: [201,205,206,207,209,210] Accelerometer: [209] Gyroscope: [209]	Alarm: [191,208] Smartwatch: [209]	Fatigue: [170,173,174,175,178,181,182,183,186,187,188,189,190,191,192,193,200,201,202,205,206,207,208,209,210] Distraction: [193]
Int. R1→R2	CNN: [191] Bayesian NN: [208] SVM: [209]	Camera: [191,208] PPG: [208,209] Accelerometer: [209] Gyroscope: [209] Vehicular: [209]	Alarm: [191,208] Smartwatch: [209]	Fatigue: [191,208,209]
Int. R2→R1				
Prob. R3	SVM: [168,173]	ECG: [173] PPG: [174] Infrared: [193] Camera: [193]		Fatigue: [168,173,174,193] Distraction: [193]
Detect R2→R3				
Int. R2→R3				
Prob. Rec.				
Int. R3				

**Table 8:** Construction AI/OSH algorithm, sensor and actuator research organized by the REDECA framework. Major technologies described by each paper is mentioned and linked to the relevant papers [211,212,213,214,215,216,217,218,219,220,221,222,223,224,225,226,227,228,229,230,231,232,233,234,235,236,237,238,239,240,241,242,243,244,245,246,247,248,249,250,251,252,253,254,255,256]. Summary of each paper is found in Appendix E.

REDECA Component	AI Algorithms (ML)	Sensors	Actuators	Environment (Type of Hazard)
Prob. R2	SVM: [254]	Pressure: [254] Motion: [256]		Fall: [254,256]
Detect R1→R2	ANN: [253] KNN: [255]	Accelerometer: [253] Audio: [255]		General Safety: [255] Fall: [215,253]
Int. R1→R2			BIM: [215,216,217,218,219,220,221]	General Safety: [216] Falls: [215,217,218,219,220,221]
Int. R2→R1	KNN: [255]	Audio: [255]		General Safety: [255]
Prob. R3	ANN: [253]	Accelerometer: [253]		Fall: [253]
Detect R2→R3	ANN: [225,248,253] KNN: [225,231,255] RBF: [225] PPCA: [225] LDA: [225] High level fuzzy: [226] Petri net: [226] GMM: [226] HMM: [245,246] SVM: [227,228,229,241,248] Decision tree: [230,232,233,234,235,244,251] Computer Vision: [238] Naïve Bayes: [242] Pattern matching: [243] Markov Chain: [247]	Accelerometer: [225,226,227,228,229,230,234,238,251] Gyroscope: [231,232,233] Barometer: [232,233] Electromyography: [235,236] Camera: [238] Vibration: [241,242,243,244] Audio: [242,243,244,245,246,247,248,249,250,251,252,253,254,255] Pressure: [245] Ambient: [246,247,248]		Fall: [225,226,227,228,229,230,231,232,233,234,235,236,238,241,242,243,244,245,246,247,248,251] General Safety: [255]
Int. R2→R3				
Prob. Rec.				
Int. R3	KNN: [255]	Audio: [255]		General Safety: [255]

## Appendix A

**Table A1:** Reports of AI Applications in the Agricultural Industry.

Study	AI Applications		Tasks	OSH Relevance or Outcome
	Sensor/ Robot	Algorithm/Technology		
[43]	Robotics	NA	Introduction of robotics for land preparation	Reducing the manual work, Prevention of musculoskeletal disorders (MSDs) which is caused over time by repetitive works
[55]	Robotics	NA	Melon detection autonomous	Reducing the manual work while detecting the melon faster
[81]	LiDAR sensors	NA	Tracking robot position from the workers	Reducing possible injuries of the workers due to the HRI
[87]	Robot sprayer	NA	Semiautomatic teleoperation of HRI system	Reducing possible injuries due to the HRI, Reducing the manual work, Preventing diseases due to the exposure to the toxic pesticides
[90]	HRI	NA	Detection of fatigue, workload, and awareness for the human operator	Preventing possible injuries due to fatigue and over workload
[91]	Agricultural robot sprayer	NA	Situation awareness for operator and robot	Reducing possible injuries due to the HRI, Reducing the manual work, Preventing diseases due to the exposure to the toxic pesticides
[94]	NA	DL, simulation	Simulate steering a tractor with almost the same accuracy as the manual steering by analyzing EMG	Reducing manual work, preventing MSDs due to the repetitive steering
[96,97,98]	Triaxial sensors	NA	Monitoring the level of exposure of the workers to vibration and repetitive activities.	Prevention of MSDs which is caused over time by repetitive works

## Appendix B

**Table A2:** Reports of AI Applications in the Oil and Gas Industry.

Study	AI Applications		Tasks	OSH Relevance or Outcome
	Sensor/ Robot	Algorithm/Technology		
[100]	Temperature and gas sensors	WSN technology using At mega 2560 controller	Oil well monitoring and control	Prevention of the workers from exposure to high-pressure line and equipment, extreme temperature environment, hazardous chemical, explosion, and fires
[101]	NA	ZigBee technology	Remote Monitoring and Control of the pipelines	Prevention of the workers from exposure to hazardous chemical, explosion, and fires
[102]	Gas safety system for oil drilling sites	WSN technology	Monitoring the oil drilling sites	Prevention of the workers from exposure to ergonomic related injuries such as lifting heavy items, bending, working in awkward postures, and repetitive tasks
[103]	Pressure, Temperature, Acoustic, Vibration sensors	Simulation-based on MATLAB and C++	An efficient oil and gas pipeline monitoring systems	Prevention of the workers from exposure to extreme temperature, high-pressure line and equipment, and ergonomic related injuries due to the repetitive exposure to vibrations
[104]	Flow sensors	WSN technology and game theoretic approach	Pipeline monitoring	Prevention of the workers from exposure to high-pressure line and equipment, hazardous chemical, explosion and fires
[105]	Pressure and temperature sensors	Simulation-based Microcontroller, Zigbee	Monitoring of oil and gas pipelines	Prevention of the workers from exposure to high-pressure line and equipment, hazardous chemical, extreme temperature environment, explosion, and fires
[106]	Pressure, Temperature, sensors	WSN technology	Monitoring of oil and gas pipelines	Prevention of the workers from exposure to high-pressure line and equipment, hazardous chemical, extreme temperature environment, explosion, and fires
[107]	Pressure transducers	SVM, KNN, GMM algorithms	Leakage detection and size estimation	Prevention of the workers from exposure to high-pressure line and equipment, hazardous chemical, extreme temperature environment, explosion, and fires
[108]	Ultrasonic transducers, flow sensors, Transit-Time Ultrasonic Flow Meter(TTUF), Doppler Ultrasonic Flowmeter(DUF)	WSN technology	Detection of leaking in long pipelines	Prevention of the workers from exposure to hazardous chemical, explosion, and fires
[109]	Pressure transducers	SVM, KNN, and GMM algorithms	Leakage detection	Prevention of the workers from exposure to high-pressure line and equipment, hazardous chemical, explosion, and fires
[110]	Magnetic induction-based, pressure and Acoustic sensors	WSN technology	Monitoring underground pipeline	Prevention of the workers from exposure to the high-pressure line and equipment, and hearing injuries
[111]	Pressure, Temperature, Acoustic sensors	WSN technology	Monitoring Underwater pipelines	Prevention of the workers from exposure to extreme temperature, high-pressure line and equipment, and hearing injuries
[112]	NA	graded network, GPRS, Anko-TC series, OMNet++	Remote monitoring terrestrial petroleum pipeline cathodic protection system	Prevention of the workers from exposure to hazardous chemical, explosion, and fires
[113]	Acoustic sensor	SVM, Wavelet Transform technology	Hierarchical leak detection and localization method in natural gas pipeline monitoring	Prevention of the workers from hearing injuries
[115]	Pressure, Temperature, Acoustic, Vibration sensors	WSN technology	Autonomously monitor and detect any defects in the different downstream operations	Prevention of the workers from exposure to extreme temperature, high-pressure line and equipment, and ergonomic related injuries due to the repetitive exposure to vibrations
[116]	Sensor nodes on the machine, Vibration, and stator current	WSN technology	Autonomously monitor and detect any defects in the operations	Prevention of the workers from ergonomic related injuries due to the repetitive exposure to vibrations
[118]	Propane sensors	WSN technology, Localization algorithms	A gas leak detection and localization with a detection rate of 91%	Prevention of the workers from exposure to hazardous chemical, explosion, and fires
[119]	Pressure, Temperature, Acoustic flow rate sensors	WSN technology	Using pipeline monitoring system to detect and localized leakage and blockage in oilfield pipelines	Prevention of the workers from exposure to extreme temperature, high-pressure line and equipment, and hearing injuries
[120]	Gas, wind, temperature, and humidity sensors	WSN technology	features from environmental sensors such as wind speed used to develop a real-time and large area wireless monitoring system for gas leakage	Prevention of the workers from exposure to high-pressure line and equipment, hazardous chemical, explosion and fires, extreme temperature and humidity environments
[120]	NA	IoT	Data collection	Prevention of the workers from exposure to extreme environments, fall sites
[122]	Different sensors in wearable watches, smart helmets, and smart glasses	IoT	Used by oil field engineers in offshore fields for real-time assistance, safety, and communication with the control room for navigation and enhanced collaboration	Prevention of the workers from exposure to extreme temperature, high-pressure line, and equipment, hazardous chemical, explosion and fires, vehicle accidents, falls, working the confined space, and ergonomic related injuries such lifting heavy items, bending, working in awkward postures
[123]	Sensor-based Pipeline Autonomous Monitoring and Maintenance System (SPAMMS)	WSN technology	Active and corrective monitoring and maintenance of the pipelines	Preventing the workers from exposure to hazardous chemical, explosion, and fires
[124]	Pressure, temperature, acoustic, vibration sensors	Radio-frequency identification (RFID	Detect anomalous events such as pipeline leakage detection	Prevention of the workers from exposure to extreme temperature, high-pressure line and equipment, and ergonomic related injuries due to the repetitive exposure to vibrations
[125]	Autonomous SystemsWireless Sensor Networks	WSN technology	Digitization of oil fieldsreal-time optimization of drilling operationsthe use of nanotechnologyto aid gauging, reservoir modeling, and diagnostics	Prevention of the workers from exposure to extreme environments, vehicle accidents, falls, working the confined space, ergonomically related injuries such lifting heavy items, bending, working in awkward postures
[126]	Smart robots	AVA classification as an unsupervised ML algorithm	Exploration of oil and gas autonomously	Prevention of the workers from exposure to extreme environments falls
[131]	NA	RF and Landsat 8 OLI imagery algorithms	Map land oil spills	Prevention of the workers from exposure to hazardous chemical, explosion, and fires
[132]	Pressure, Temperature, Acoustic, Vibration sensors	LS-SVM machine learning algorithm, acoustic wave method	Detect leak levels on a gas pipeline	Prevention of the workers from exposure to high-pressure line and equipment, extreme temperature environment, hazardous chemical, explosion, and fires
[133]	Autonomous robot	NA	Inspection of pipeline and other equipment	Prevention of the workers from exposure to extreme environments, vehicle accidents, falls, working the confined space, ergonomic related injuries such as lifting heavy items, bending, working in awkward postures

## Appendix C

**Table A3:** Reports of AI Applications in the Mining Industry.

Study	AI Applications		Tasks	OSH Relevance or Outcome
	Sensor/ Robot	Algorithm/Technology		
[137]	NA	Decision tree, RF, NN	Predicting mining accident and days away from work	Preventing from accidents in the mining industry by predicting it
[139]	GPS	NA	Monitoring locations and movements	Preventing from fall injury by measuring the gait stability, estimating the fall risk, preventing from entering the workers to the hazardous environments by identifying the location of a workers and warning
[139]	Motion and speed sensors, Communication sensors	NA	Monitor the movement of the workers in remote locations and communicate with them	Preventing from a fall injury, ensuring the safety of the workers by staying connected and communicating with them while they are in the mine
[143]	Environmental sensors	NA	Collecting data from surrounding of the workers	Preventing the workers from harmful bacteria due to high level of humidity, high temperature, hearing damage, and toxic gases in the mine
[144]	Toxic gases sensors	NA	Monitoring the level of the toxic gases	Protection of the workers from exposure to the toxic and inflammable gases and prevention of fire explosion in the mine for their safety
[146]	Acoustic sensors, and wearable dust sensors	NA	Monitoring the noise level and monitoring the level of exposure to crystalline silica	Preventing hearing damage and hearing loss of the workers due to the high level of noise in the mine and prevention of respiratory diseases due to the exposure to crystalline silica over time
[147]	IoT and smart robots	NA	Monitoring the real-time information of the mine and mineworkers	Exploring inaccessible areas underground which hazardous situations occur with unpredictable risks that are too severe for human activity
[152]	Smart Helmet Clip wearable sensor	NA	Monitoring the surrounding of the workers to identify the presence of the dangerous gasses	Prevention of the exposure of the workers from toxic and inflammable gases, fire, and explosion in the mine for the safety of the workers
[153]	Angel helmet	NA	Monitoring location and positioning of the workers	Preventing from fall injury by measuring the gait stability, estimating the fall risk, preventing from entering the workers to the hazardous environments by identifying the location of a workers and warning
[154]	Safety helmet with CH4 and CO gas sensors	NA	Monitoring the level of the CH4 and CO gases	Protection of the workers from exposure to the CH4 and CO which are toxic and inflammable in the mine for their safety
[155]	Helmet-Cam	NA	Monitoring the concentration of silica dust	Prevention of respiratory diseases due to the exposure to crystalline silica over time
[156]	Smart devices combining a safety vest, eyewear, helmet, and watch	NA	Monitoring the health and safety of workers in different aspects and monitoring activity of the workers to prevent fall injury	Preventing the workers from head and body injuries through wearing smart eyewear, safety vest, and helmet, preventing the workers from high temperature, hearing damage, and toxic gases in the mine through the smartwatch, and preventing from fall injury of the worker at the mine
[157]	Smartwatch	NA	Monitoring the motion and health of the workers	Preventing from fall injury by measuring the gait stability, estimating the fall risk, preventing from entering the workers to the hazardous environments by identifying the location of a workers and warning
[158]	Smartwatch	NA	Distinguishing the normal from the abnormal posture of the workers	Prevention of the fall injures and reducing risks for MSDs which is caused over time by repeating the abnormal posture of the workers in the mine

## Appendix D

**Table A4:** Reports of AI Applications in the Transportation Industry.

Study	AI Application		Task
	Sensor/ Robot	Algorithm/Technology	
[173]	non-invasive sensors embedded in steering wheels	SVM	Fatigue detection through analyzing ECG features
[174]	PPG sensor on the steering wheel	SVM	Fatigue detection through analyzing PPG features
[175]	Computer vision Sensors on the forehead	SVM	Fatigue detection by analyzing the EoG features
[176]	EEG sensors	Bayesian neural network	Assessment of mental workload, detection of fatigue and drowsiness by analyzing EEG features
[179]	sEMG	Power in band	Fatigue detection by analyzing the sEMG features
[181]	ECG, EEG, EoG	Digital signal processing	Fatigue detection by analyzing the ECG, EEG, EoG features
[184]	Computer vision	Various ML models	Fatigue detection by analyzing the mouth, eye, head movements, and facial expressions as features
[186]	Computer vision	Various ML models	Fatigue detection by analyzing the face tracking method
[188]	Computer vision	SVM	Fatigue detection by analyzing the mouth movement features
[189]	Computer vision	DL	Fatigue detection by analyzing the mouth movement features
[199]	Computer vision	RF	Detecting fatigue through analyzing steering wheel angles
[200]	Computer vision	Binary decision classifier	Detection of fatigue through analyzing the steering wheel angles as features
[203]	Driving simulator	GMM and the Helly model	Fatigue detection by analyzing vehicle velocity, brake pedal, accelerator pedal, and distance from car in-front as features
[204]	Multiple onboard sensors	Linear discriminant	Fatigue detection through analyzing both driver characteristics and vehicle characteristics
[205]	Computer vision, vehicle movement sensors	two-stage data fusion framework	Fatigue detection by analyzing driver and vehicle characteristics
[206]	Computer vision, vehicle movement sensors	ANN	Fatigue detection by analyzing the physiological and behavioral features of the driver
[207]	Computer vision, vehicle movement sensors	M-SVM	Fatigue detection by analyzing the combinations of biological, and vehicular features

OSH relevance or outcome in all cases is ‘preventing accident due to fatigue’.

## Appendix E

**Table A5:** Reports of AI Applications in Wearable Devices in Construction.

Study	AI Application	
	Sensor/Robot	Algorithm/Technology
[224]	Accelerometer	Comparator system
[226]	Accelerometer	High level fuzzy, Petri net, GMM
[228]	Accelerometer	SVM
[229]	Accelerometer	Decision tree
[231]	Accelerometer, gyroscope	k-NN
[232]	Accelerometer, gyroscope, barometric altimeter	Decision tree
[233]	Accelerometer, barometric pressure, Gyroscope	Decision tree
[234]	Accelerometer, cardio tachometer	Decision tree
[235]	Electromyography	Decision tree
[240]	Smartphone	Decision tree
[241]	Vibration	SVM
[242]	Vibration, microphone	Naïve Bayes
[243]	Special Piezo pressure transducer	Pattern matching
[244]	Special Piezo pressure transducer	Decision tree
[245]	Special Piezo pressure transducer	HMM

OSH relevance or outcome in all cases is ‘detection and prevention of fall’. Tasks in all cases are ‘Detecting fall through ML algorithm by analyzing the data which collected through sensors’.
